# Supplementary material for: Causal interplay between lactose intolerance and gut microbiota: a combined bidirectional Mendelian randomization and in vivo validation study
Source: Front Nutr. 2026 Jun 1;13:1803337. doi: 10.3389/fnut.2026.1803337 (PMC13265576; doi:10.3389/fnut.2026.1803337)
Supplement: Supplementary file 1 [file Data_Sheet_1.zip › supplementary materials/Forward/leave one out cross validation/ebi-a-GCST90027635.finngen_R12_E4_LACTONAS.pdf]

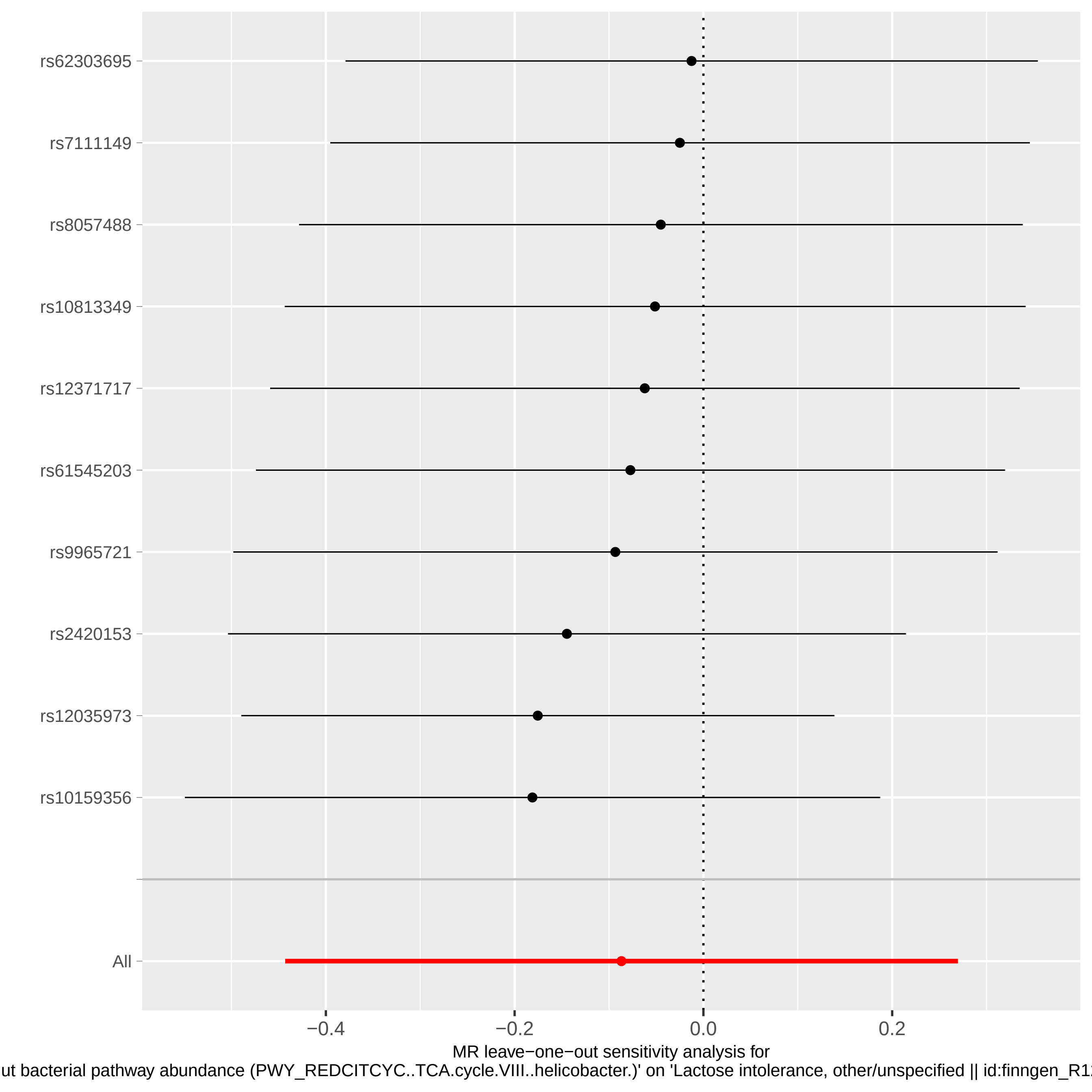
rs62303695
rs7111149
rs8057488
rs10813349
rs12371717
rs61545203
rs9965721
rs2420153
rs12035973
rs10159356
All
−0.4
−0.2
0.0
0.2
MR leave−one−out sensitivity analysis for
ut bacterial pathway abundance (PWY_REDCITCYC..TCA.cycle.VIII..helicobacter.)' on 'Lactose intolerance, other/unspecified || id:finngen_R1
